# When stress drives you to kidnap: RACK1A sequesters FSD1 into stress granules during salt stress

**DOI:** 10.1093/plphys/kiaf673

**Published:** 2026-01-06

**Authors:** Sara Selma

**Affiliations:** Assistant Features Editor, Plant Physiology, American Society of Plant Biologists, Rockville, USA; VIB Center for Plant Systems Biology, Ghent 9052, Belgium

Describing plants as sessile organisms may sound like a scientific revelation, but it simply means they have nowhere to go. However, it serves as a fitting reminder of how crucial it is for them to have successful defense mechanisms against environmental threats to ensure their static survival. One of the biggest harms that plants face is salinity stress, which involves complex signaling networks and second messengers, such as reactive oxygen species (ROS), to mitigate it (Zhou et al. 2024). ROS are fantastic signal molecules to wake up the plant's defense, but they can also be harmful to the plant when they accumulate in excess (Wang et al. 2024). This duality requires plants to possess a fine-tuned capacity to keep the homeostasis of ROS molecules. An enhanced capacity to regulate ROS levels is also linked to better tolerance and survival during salt stress. Superoxide dismutases (SODs) are frontline ROS-detoxifying enzymes that keep superoxide radicals (very reactive oxidative molecules) under control by converting them into hydrogen peroxide (Kliebenstein et al. 1998). Iron Superoxide Dismutase 1 (FSD1) plays a critical role in maintaining ROS balance, especially during salt stress (Dvořák et al. 2021). Despite its importance, the regulatory mechanism of FSD1 during salt stress is still not fully understood.

Interestingly, FSD1 was found in a large-scale yeast 2-hybrid screen as an interacting partner of RECEPTOR FOR ACTIVATED C KINASE 1 A (RACK1A), a cytoplasmic protein that acts as a scaffold in signaling pathways during stress responses and development (Guo et al. 2011). Although the RACK1 family had been linked to the regulation of salt stress in plants, their role is unclear and sometimes contradictory, depending on the plant species (Li et al. 2017; Zhang et al. 2018).

Melicher and colleagues delved into the role of FSD1-RACK1A interaction in the context of plant salt stress in *Arabidopsis thaliana* (Arabidopsis). First of all, the analysis of the expression of the RACK1A, employing a complementation line of *rack1a* with the *proRACK1A::RACK1A:GFP* construct, showed that the RACK1A-GFP is expressed in the cytoplasm but with preference in meristematic regions of roots, suggesting a role in the root hair formation and root growth. Additionally, RACK1A showed the same expression pattern as FDS1, reinforcing the physiological relevance of the FSD1-RACK1A interaction, which was also validated by both salt-induced GFP-trap-MS interactome analysis and biomolecular fluorescence complementation assays. The salt-induced RACK1A interactome also showed other SOD proteins and the proteins TUDOR STAPHYLOCOCCAL NUCLEASE (TSN) 1 and 2. The TSN2-RACK1 interaction was previously reported for RACK1B and was involved in the formation of stress granules (SGs), which are protein aggregates triggered by stress situations to inhibit translation and protect proteins from unfolding or degradation (Gutierrez-Beltran et al. 2021). The rBiFC experiments further confirmed that the FSD1-RACK1A interaction is largely cytosolic, with minimal nuclear localization. Additionally, the interaction TNS1-RACK1A was also confirmed in the cytosol, pointing to its role as a cytosolic scaffold of SGs during stress responses.

To assess the physiological relevance of the FSD1-RACK1A interaction, double mutants and single mutant lines for *rack1a* and *fsd1* were evaluated for their total SOD activity. Surprisingly, both *rack1a* and *rack1a-fsd1* mutants showed an enhanced SOD activity compared with wild type (WT). However, the enhanced SOD activity was not appreciated in the single *fsd1* mutant, suggesting that SOD regulation is through RACK1A. Additionally, in *rack1a* mutants, although the activity of all the SOD isozymes was higher than in WT, the protein abundance of FSD1 remains unchanged; this is contrary to the other SOD evaluated, which showed an increased protein abundance. This points to the activity of FSD1 being modulated by the direct interaction with RACK1A. Finally, in vitro assays with recombinant RACK1A showed a reduction of total SOD activity in WT and rack1a-1 extracts but not in fsd1-1, confirming that RACK1A negatively regulates FSD1 activity but also influences the overall SOD activity through its interaction with FSD1.

The functional analysis further confirms the influence of RACK1A and FSD1 on root hair development. Consistent with this, *rack1a* mutants and *rack1a-fsd1* double mutants display altered primary root growth. Notably, the double mutant exhibits the shortest roots, indicating an additive genetic effect of FSD1 and RACK1A.

Most interestingly, salt stress conditions trigger the formation of cytosolic protein condensates of RACK1A, which are absent under control conditions and disappear upon recovery or in the presence of the SG inhibitor cycloheximide. These RACK1A-containing condensates colocalize strongly with the SG marker RBP47 fused to RFP, confirming that RACK1A aggregates in SGs in response to salt stress. Similarly, FSD1 colocalizes with RACK1 in the formation of SGs under salt stress conditions but fails in the rack1a mutant line, suggesting that RACK1A is required for FSD1 to be recruited to SGs during salt stress.

To analyze what is happening in those SGs, the authors evaluated FSD1 activity in an SG–enriched fraction, revealing that its enzymatic activity is lower in the SGs than in the soluble cytoplasmic pool under salt stress. Consistently, ROS analyses and salt tolerance assays show that *rack1a* mutants are more resistant to salt stress, *fsd1* mutants are more sensitive, and the double mutant exhibits an intermediate phenotype, supporting a joint role of RACK1A and FSD1 in modulating ROS levels during the salt stress response.

In sum, Melicher et al. show a model in which the FSD1-RACK1A interaction fine-tunes ROS homeostasis by kidnapping and inactivating FSD1 in SGs under salt stress conditions (Fig. 1). Additionally, under normal growth conditions, the interaction FSD1-RACK1A could promote root hair elongation, regulating the availability of ROS in the meristems. This study illustrates how plants employ multilayered regulatory mechanisms to balance growth and defense and to ensure they survive and thrive.

**Figure 1.**
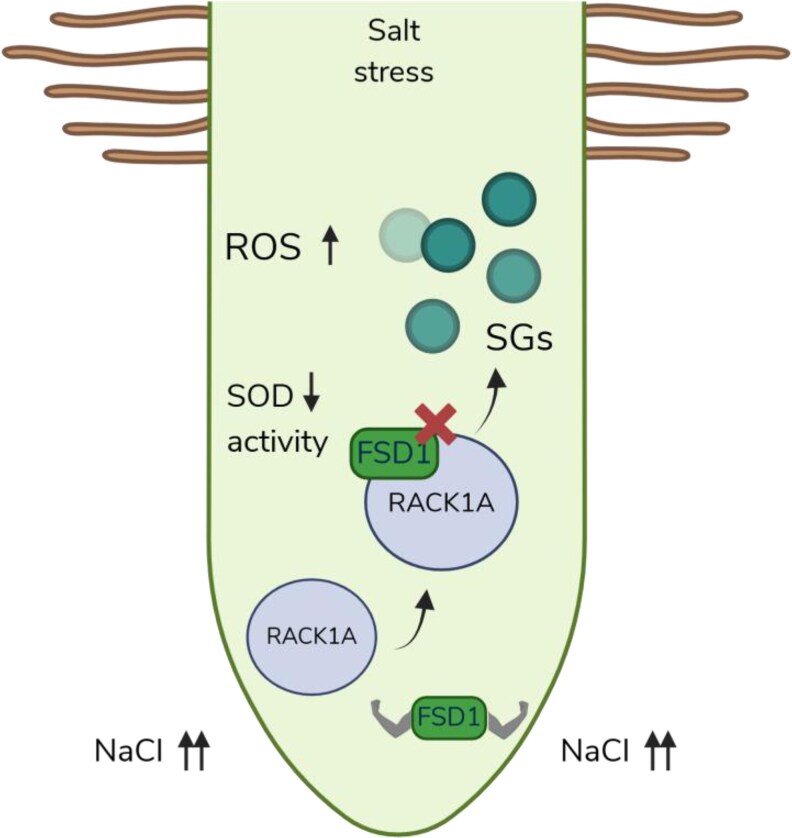
A Mechanistic model of FSD1-RACK1A interaction under salt stress. In the root meristems, under salt stress (NaCl), the protein RACK1A interacts with FSD1. RACK1A kidnaps FSD1 in SGs, reducing the SOD activity of FSD1 in the cell and thus allowing an increase in ROS. Image created by Biorender.com.

## Related publications

Jianjun Guo, Shucai Wang, Oliver Valerius, Hardy Hall, Qingning Zeng, Jian-Feng Li, David J. Weston, Brian E. Ellis, Jin-Gui Chen, Involvement of Arabidopsis RACK1 in Protein Translation and Its Regulation by Abscisic Acid, Plant Physiology, Volume 155, Issue 1, January 2011, Pages 370–383, https://doi.org/10.1104/pp.110.160663.

Daniel J. Kliebenstein, Rita-Ann Monde, Robert L. Last, Superoxide Dismutase in Arabidopsis: An Eclectic Enzyme Family with Disparate Regulation and Protein Localization, *Plant Physiology*, Volume 118, Issue 2, October 1998, Pages 637–650, https://doi.org/10.1104/pp.118.2.637.

Gerrit West, Dirk Inzé, Gerrit T.S. Beemster, Cell Cycle Modulation in the Response of the Primary Root of Arabidopsis to Salt Stress, *Plant Physiology*, Volume 135, Issue 2, June 2004, Pages 1050–1058, https://doi.org/10.1104/pp.104.040022.
